# Tibial Bone-Anchored Prostheses: A Narrative Review of the Literature and Reflection on 10 Years of Surgical Practice

**DOI:** 10.1007/s10439-026-04065-1

**Published:** 2026-03-07

**Authors:** R. Atallah, JP. Frölke, R. Leijendekkers

**Affiliations:** 1 Department of Orthopaedics, The Sint Maartenskliniek, Nijmegen, The Netherlands; 2 Department of Surgery, Radboud University Medical Center, Nijmegen, The Netherlands; 3 Department of Rehabilitation, Radboud University Medical Center, Nijmegen, The Netherlands

**Keywords:** Amputation, Bone-anchored prosthesis, Osseointegration, Implant design

## Abstract

**Purpose of Review:**

Historically, bone-anchored prosthesis (BAP) treatment was developed for the treatment of individuals with a transfemoral amputation. However, increasing experience with osseointegration implant (OI) surgery has resulted in its use in individuals with a transtibial amputation. In this article, we describe the emergence and evolution of BAP treatment for individuals with a transtibial amputation, review the current literature, and report on 10 years of clinical experience at our center.

**Recent Findings:**

The first tibial OI cases experienced high failure rates due to infection and aseptic loosening. However, all the following published studies reported improvements to functional outcomes and low rates of major complications. In the past, there was a clear lack of fixed follow-up, longer term studies, and stratification of outcomes. At our center, we have treated a total of 124 patients with 133 tibial BAPs over the course of 10 years, starting in 2014, and followed up for a mean of 58 months, ranging from 9 to 131 months. Five different OI designs were used over this period, showing the evolution of design philosophies and adaptations to challenges over time. A total of 11 OIs failed/were removed for reasons such as implant breakage (2), chronic knee pain (2), early infection (3), late infection (2), failure of osseointegration (2).

**Summary:**

Tibial BAP treatment has proven increasingly safe with growing patient demand. Initial fears about failure of fixation/loosening seem unfounded due to optimization of the designs. Our current aim is to improve implant survival and to answer questions about: optimal patient selection, minimum area of osseointegration necessary, need for additional screw fixation, and optimal level of implant placement.

## Introduction

Bone-anchored prostheses (BAPs) using an osseointegration implant (OI) have now been used as an alternative to socket-suspended prostheses (SSP) for decades [1, 2]. After building on the discovery of the phenomenon of osseointegration, Prof. Rickard Brånemark extrapolated this knowledge used for the treatment of the edentulous jaw to treat individuals with a transfemoral amputation (TFA) [1, 3, 4]. The osseointegrated prostheses for the Rehabilitation of Amputees (OPRA) implant system was used for the first time in 1990 with no standardized rehabilitation or surgical technique protocol[5]. This initial experience ultimately led to adaptations in implant design, surgical technique, and changes in the rehabilitation protocol in 1999 [2, 5, 6]. Initial use of the OPRA implant system occurred mainly in individuals with a TFA, and Food and Drug Administration approval (which was finally established in 2019), only permitted its use in individuals with a TFA [7].

The development of the first press-fit implant system occurred in 1999 in Germany, by Dr. H. Aschoff and Dr. H. Grundei. The Integral Leg Prosthesis (ILP) was made of a Cobalt–Chrome–Molybdenum (Co–Cr–Mo) alloy with a 1.5 mm thick tripod-like structure of trabecular metal covering the intramedullary stem. The implant also had a slight curve to follow the femoral antecurvation, and was designed primarily for the use in TFA [8–10]. The success of BAP treatment in individuals with a TFA ultimately led to its first use in individuals with a transtibial amputation (TTA) in 2009, and the first report of outcomes in 2015 by Juhnke et al. [11]. The fact that it took several decades for the first BAP to be used in individuals with a TTA is best explained by the prevailing opinion that individuals with a TFA experience more difficulties with their SSP compared to individuals with a TTA [12]. Moreover, achieving stable implant fixation in the tibial metaphysis (which is short, funnel shaped, and has a drop-like cross-section for the intramedullary cavity)was thought to be more difficult to achieve. Especially when compared to the long, circular cross-section of the intramedullary cavity of a mid-diaphyseal TFA [13]. These morphological differences increased concerns among surgeons about OI fixation in the tibia, while also making it necessary to use custom made OIs, making BAP treatment at this level more complex and expensive. These anatomical challenges are also likely to have contributed to the relative absence of cases using a screw-type implant design in individuals with a TTA.

Further, there was uncertainty about the functional requirements of transtibial BAP treatment and (coupled with the poor initial outcomes) this led to a general reluctance to pursue this form of treatment [14]. However, an indication for tibial BAP treatment certainly exists, since the prevalence of TTA is similar to that of TFA in the United States and even greater in the Netherlands [15–17]. Furthermore, individuals with a TTA using an SSP experience dermatological issues just as frequently, with Dudek et al. even suggesting that this is 4 times more likely compared to individuals with a TFA [18]. Dissatisfaction rates with a SSP also show no difference comparing individuals with a TFA and TTA ranging from 33 to 57% [19, 20]. Finally, the fact that individuals with a TTA also wish to undergo BAP treatment is confirmed by the steady increase in the number of referrals to our center over the last decade [21, 22].

Therefore, the aim of this paper is to describe the emergence and evolution of tibial BAP over time. This is done through a narrative overview of the current literature and a report on 10 years of clinical experience at our treatment center. Furthermore, opinions and recommendations based on experience are also provided (i.e., not data-driven).

### Current Literature

To date there have been no longitudinal studies reporting either functional or complication outcomes after the use of the OPRA implant system in individuals with a TTA. There have been only two reports of a total of four individuals treated with a screw-type tibial OI [23, 24].

As previously mentioned, the first report on the use of press-fit OI’s in individuals with a TTA occurred in 2015 by the German group, led by Dr. Aschoff [11]. Seven individuals with a TTA were treated with 9 CoCrMo implants, mostly having a short TTA well above the isthmus. The authors did not report on implant details. However, they did report on increasing the depth of the superficial tripod structure from 1.5 to 2.5 mm due to potential worries about primary stability. They also reported on a prolonged waiting period between step 1 surgery (implantation), and step 2 surgery (stoma formation), to allow osseointegration to occur, ultimately delaying rehabilitation. Lastly the option of using transverse locking screws to counteract rotational forces was mentioned as a suggestion. However, it is unclear if transverse locking screws were used in this subcohort [11]. The German research team eventually reported on the outcomes of this subcohort, describing a 33% rate (3 out of 9) of (a)septic implant loosening, and a 44% rate (4 out of 9) of explantation [25, 26].

A new wave of studies followed these initial reports all of which reporting on the use of press-fit titanium OIs. These studies described the OI and BAP treatment outcomes in more detail. However, all studies were compromised by small sample sizes, and/or a lack of fixed periods of follow-up [27–36]. The first was a case series by Atallah et al. involving a collaboration between the Dutch and Australian group, published in 2017 reporting on tibial BAP treatment of five individuals with a history of peripheral vascular disease who were followed for 12 months [28]. The press-fit OIs used for these cases were based on a computed tomography (CT) scan and were 3D printed from titanium with a lattice structure coating. They included one or more proximal transverse screws for additional primary and rotational stability (design 2, see Table 1). No major adverse events occurred, and all the patients were using their BAP at follow-up, showing improvements in prosthetic use, walking ability, and physical aspects of quality of life. Since then, multiple small cohort studies and one large cohort study have been published reporting on individuals treated with tibial BAPs, with a maximum of 1–2 years follow-up [29, 31, 32, 34–37]. We found three other studies of tibial BAP treatment with varying follow-up periods between 2 and 6 years reporting on several extraordinary cohorts, such as burn victims, and amputations due to sepsis or peripheral vascular disease. However, as with the previous studies, these included small cohorts (< 10 patients) [27, 30, 33]. Importantly, the outcomes of all of these previous studies have been generally favorable with regards to functional improvement and safety. However, it is also notable that there were large differences in surgical protocols (including both on-stage and two-stage surgery), and rehabilitation protocols, (especially regarding loading regimen) which changed over time in the different surgical centers reporting their outcomes.

**Table 1.** Implant design data

Implant system	Material	Fabrication	Surface	Distal drop shape	Rotational stability
1	Cobalt–chrome–molybdenum	Cast	1.5 mm tripod-like trabecular metal	No	No, slight stem antecurvation
2	Titanium alloy (Ti-6Al-4 V)	3D electron beam Melting	2 mm 3D lattice structure proximal, titanium plasma spray distal	No, flared distal portion	Distal fins + distal flared triangular portion
3	Titanium (Ti-6Al-4 V)	3D Direct metal laser sintering + HIP	1 mm 3D lattice structure	Yes, grit-blasted	Locking screw options + distal drop shape
4	Titanium (Ti-6Al-4 V)	3D Direct metal laser sintering + HIP	1 mm 3D lattice structure	Yes, coated with 3D lattice structure	Locking screw options + distal drop shape
5	Titanium (Ti-6Al-7Nb)	CNC milling	0.35 mm titanium plasma spray	Yes, coated with titanium plasma spray	Locking screw options + distal drop shape

*3D* 3-dimensional, *mm* millimeters, *HIP* hot isostatic pressing

A recently published cohort study with a 5 years follow-up is the only study reporting on tibial BAP treatment at a fixed midterm follow-up to this date, reporting on a subcohort with 22 tibial BAPs [21]. A more standardized press-fit titanium OI was used with similar properties to the previously mentioned OI. However, the implant design also included a drop-like distal extension to seal of the intramedullary canal (design 3, see Table 1). A one-, two-, and five-year implant survival of 98.9%, 98.9%, and 95.5%, respectively, was reported, along with substantial increases in prosthetic use and quality of life.

No long-term fixed follow-up studies have been published to date. However, Hoellwarth et al. reported on a large cohort of individuals treated with tibial OIs with a follow-up ranging from months to eight years [38]. They did not investigate implant survival but analyzed death due to any cause, concluding that patients receiving BAP treatment rarely die of complications related to treatment.

### Chronology of Implant Design Used at our Center

Table 1 summarizes the five consecutive OI designs used for patients treated at our center from the start of the first tibial BAP treatment in 2014, until the end of 2024 (Table 1). A total of 120 individuals were treated with 133 implants, as nine were bilateral and four individuals with OI failure received a revision to another tibial OI within the timeframe mentioned. Detailed patient and OI characteristics are presented in Table 2. The mean follow-up of all 133 implants at the time of analysis in October 2025 was 58 months with a standard deviation of 33 months, ranging between 9 and 131 months. Eleven implants were explanted. The reasons for explantation will be given in the outcomes section. Functional outcome data can be found in previously published studies and will be made available in future publications [21, 28, 29, 31, 36, 38]. This study was approved by the local Ethics committee of the Radboud university medical center (registration nr. 2014/196).

**Table 2.** Patient and implant characteristics

	Total (*N* = 120)	Design 1 (*N* = 1)	Design 2 (*N* = 8)	Design 3 (*N* = 55)	Design 4 (*N* = 9)	Design 5 (*N* = 51)
Patient demographics						
Male sex^a^	80 (67%)	0 (0%)	5 (63%)	42 (76%)	4 (44%)	30 (59%)
Age at implantation in years^b^	50 ± 12	42	44 ± 14	52 ± 12	49 ± 9	44 ± 18
Baseline amputation characteristics						
Level^a^ (per limb: *N* = 129^c^)						
•TTA	122 (95%)	NA	8 (100%)	54 (98%)	9 (100%)	46 (90%)
Syme/Pirogoff	4 (3%)	1 (100%)	NA	NA	NA	3 (6%)
Non-amputated	3 (2%)	NA	NA	1 (2%)	NA	2 (4%)
Side^a^						
Left	58 (48%)	1 (100%)	5 (63%)	23 (42%)	5 (56%)	28 (55%)
Right	53 (44%)	NA	3 (38%)	28 (51%)	4 (44%)	18 (35%)
Bilateral	9 (8%)	NA	NA	4 (7%)	NA	5 (10%)
Cause (per patient: *N* = 120)						
Trauma	82 (68%)	1 (100%)	5 (63%)	39 (71%)	8 (89%)	31 (61%)
Dysvascular	17 (14%)	NA	2 (25%)	9 (16%)	NA	6 (12%)
Infection	11 (9%)	NA	1 (13%)	4 (7%)	NA	7 (14%)
Tumor	5 (4%)	NA	NA	NA	1 (11%)	5 (10%)
Congenital	1 (1%)	NA	NA	NA	NA	1 (2%)
Other	4 (3%)	NA	NA	3 (5)	NA	1 (2%)
Implant characteristics (per implant: N = 133)	133 implants	1 implants	8 implants	59 implants	9 implants	56 implants
Width (mm)^b^	20 [11 to 33]	11	23.5 [19 to 33]	21 [17 to 32]	18 [15 to 30]	19 [15 to 32]
Length (mm)^b^	80 [45 to 145]	105	100 [70 to 130]	90 [60 to 145]	70 [46 to 115]	80 [45 to 120]

*N* participants

^a^The values are given as the number of patients/implants with the percentage in parentheses

^b^The values are given as the mean and standard deviation

^c^The values are given as the median and range. Total of 5 designs amounts to 124 because 4 patients out of 120 were revised after failure

*TTA* transtibial amputation

#### Implant System 1

**Fig. 1.**
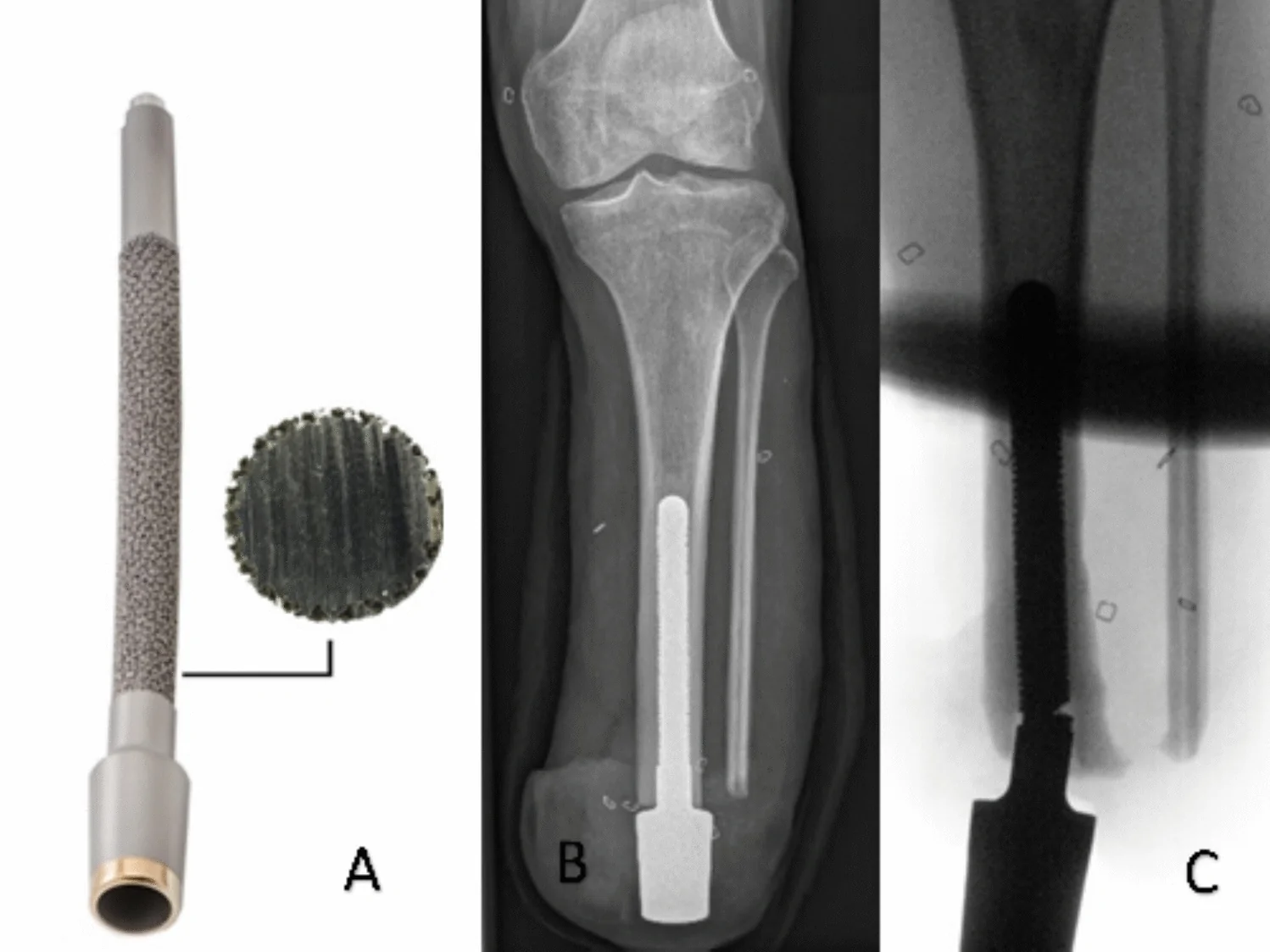
Implant system 1. **A** Photograph of Integral Leg Prosthesis design with transverse view, **B** Postoperative anteroposterior radiograph, **C** Radiograph of implant breakage at transitional level of coating

Implant system 1 was only used in 1 individual in 2014. It was a smaller copy of the Integral Leg Prosthesis used for individuals with a TFA (ILP, Orthodynamics GMbH, Lübeck, Germany) (Fig. 1A). This patient had previously undergone a Pirogoff amputation and presented with amputation-related complaints, and was ultimately treated with a slight shortening and implantation at the mid-diaphyseal level. Due to its diaphyseal location and the patient’s narrow intramedullary canal an implant with a diameter of only 11 millimeters (mm) was used (Fig. 1B). Implant breakage occurred at the distal level of transition from the coated surface after 60 months of follow-up (Fig. 1C). Implant survival and location of the site of the break corresponded to what has been previously observed in a cohort of individuals with a TFA treated with an ILP implant, especially occurring within implants with a smaller diameter (15 millimeters or smaller) [39]. The individual underwent successful reimplantation with a metaphyseal implant system four-type device.

#### Implant system 2

**Fig. 2.**
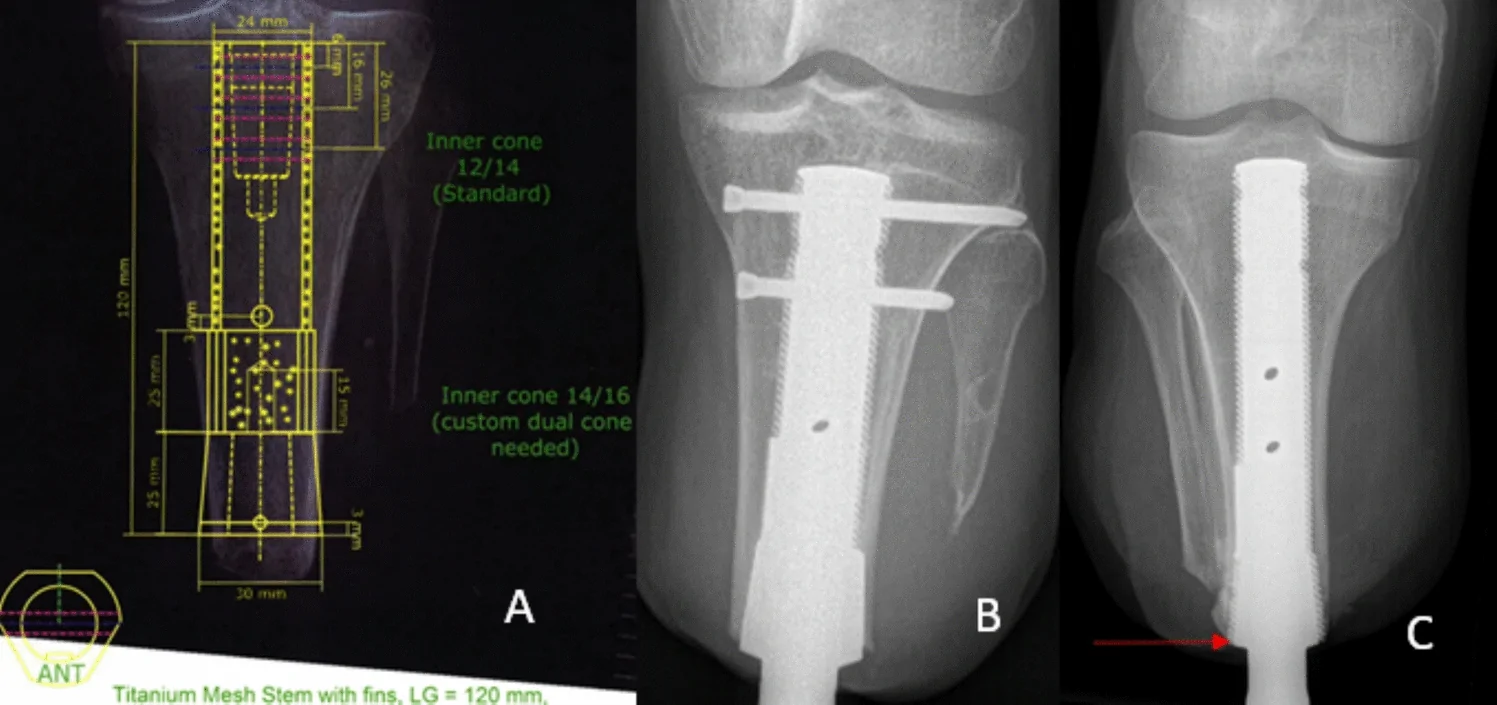
Implant system 2. **A** Planning of implant design on radiograph, **B** Anteroposterior radiograph after implantation, and **C** Radiograph of distal bone resorption with uncoverage of lattice structure at level of stoma (arrow)

Implant system 2 was used for eight individuals in the period between 2014 and 2015. It was called the Patient Specific Implant tibia OD or AQ (PSI Tibia OD/AQ) and produced by both Orthodynamics (OD: Orthodynamics GMbH, Lübeck, Germany) and AQ Implants (AQ Implants GMbH, Ahrensburg, Germany). The implant was made using 3D Electron Beam Melting technology from titanium grade 5 powder (Ti-6Al-4 V) and was covered with a 2 mm 3D lattice structure on the proximal portion and with either titanium plasma spray (TPS) or a 1 mm lattice structure on the distal portion. The implant also contained fins protruding 1 mm, a tapered distal portion and the option for proximal 5 mm transverse screw fixation (Fig. 2A, B). A few adaptations were observed when comparing the tibial design to established femoral BAP implants: 1. Increasing the surface area of osseointegration using a thicker lattice surface structure, 2. Additional primary and rotational stability achieved using distal fins and proximal transverse locking screws, 3. Specific tapered distal medullary fit and closure. All of these adaptations were intended to achieve improved implant fixation, considered the main challenge in tibial BAP. However, the distal flared portion resulted in cortical cracks in some cases, treated with cerclage wires that were later removed due to complaints. Furthermore, the distal tapered portion was also coated with a lattice structure in some cases, and when distal bone resorption occurred over time, this exposed lattice/mesh structure could result in soft tissue complaints at the level of the stoma.

#### Implant System 3

**Fig. 3.**
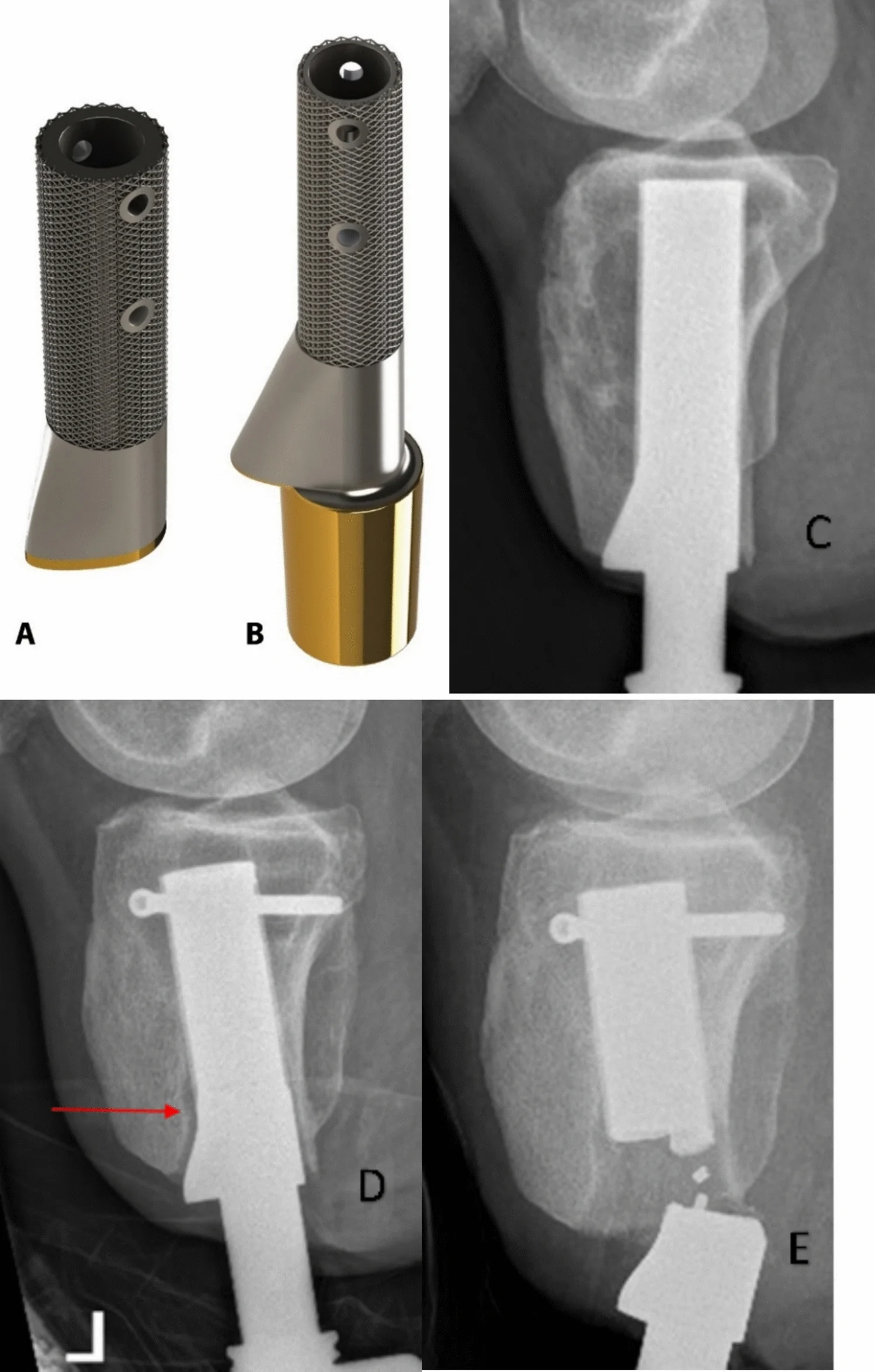
Implant system 3. A/B. 3D rendering of implant design without **A** and with **B** extramedullary head, **C** lateral radiograph at 5 years follow-up showing no radiolucent lines, **D** Radiograph showing distal radiolucent lines especially at uncoated part (arrow), **e** Radiograph showing subsequent implant breakage at transition from coating

Implant system 3 was used in 55 individuals with 59 implants in the period 2016-2020. It was called the PSI OTNi CM4 and was designed based on CT scan images and produced by OTN implants (OTN Implants, Nijmegen, The Netherlands). The implant was manufactured by Direct Metal Laser Sintering (DMLS) 3D printing technology combined with Hot Isostatic Pressing (HIP) from titanium grade 5 powder (Ti-6Al-4 V). The cylindrical implant included a distal drop-like extension to correspond to the anatomy of the tibial intramedullary cavity [13]. This distal oval/drop-like shape was calculated and standardized with a mathematical formula based on CT scans of 100 subjects (unpublished data). The proximal part of the implant was covered with a 1 mm 3D lattice structure while the distal drop was grit blasted. The lattice structure intended to enhance the “surface area for osseointegration” by a factor of 3.7 [29]. The implant contained options for 5 mm proximal transverse locking screw fixation (Fig 3A, B). In the case of implants with a distal extramedullary head, the head was mirror polished and TiNbN coated (Fig 3B). The main differences to implant system 2 included: 1. Increased standardization of design, 2. Anatomical drop-like extension aiming to seal off the intramedullary canal and providing rotational stability and decreasing the risk of soft tissue irritation in cases with distal bone resorption, 3. Decreased size of lattice structure, resulting in increased core diameter. Even with the absence of additive surface treatment (i.e., coating) on the distal portion, bony ingrowth generally occurred (Fig. 3C). However, radiolucent lines were observed over time, mostly involving the uncoated distal portion, although not usually resulting in adverse events (Fig. 3D). Possible explanations about the reasons for the radiolucent line formation, such as: 1. stress shielding/lack of osseous stimulation in absence of a coating, 2. ascending distal infectious osteitis. There was one case of implant breakage. This occurred at the transition level between the lattice structure and the distal portion (Fig. 3E). This was analyzed as a fatigue fracture at the implant level where the drop-like extension transitions to the cylindrical part. We speculated that the transition from a 3D lattice structure to the rest of the implant may also act as a stress riser from which device breakages can propagate, as was also described in the ILP implant [39].

#### Implant System 4

**Fig. 4.**
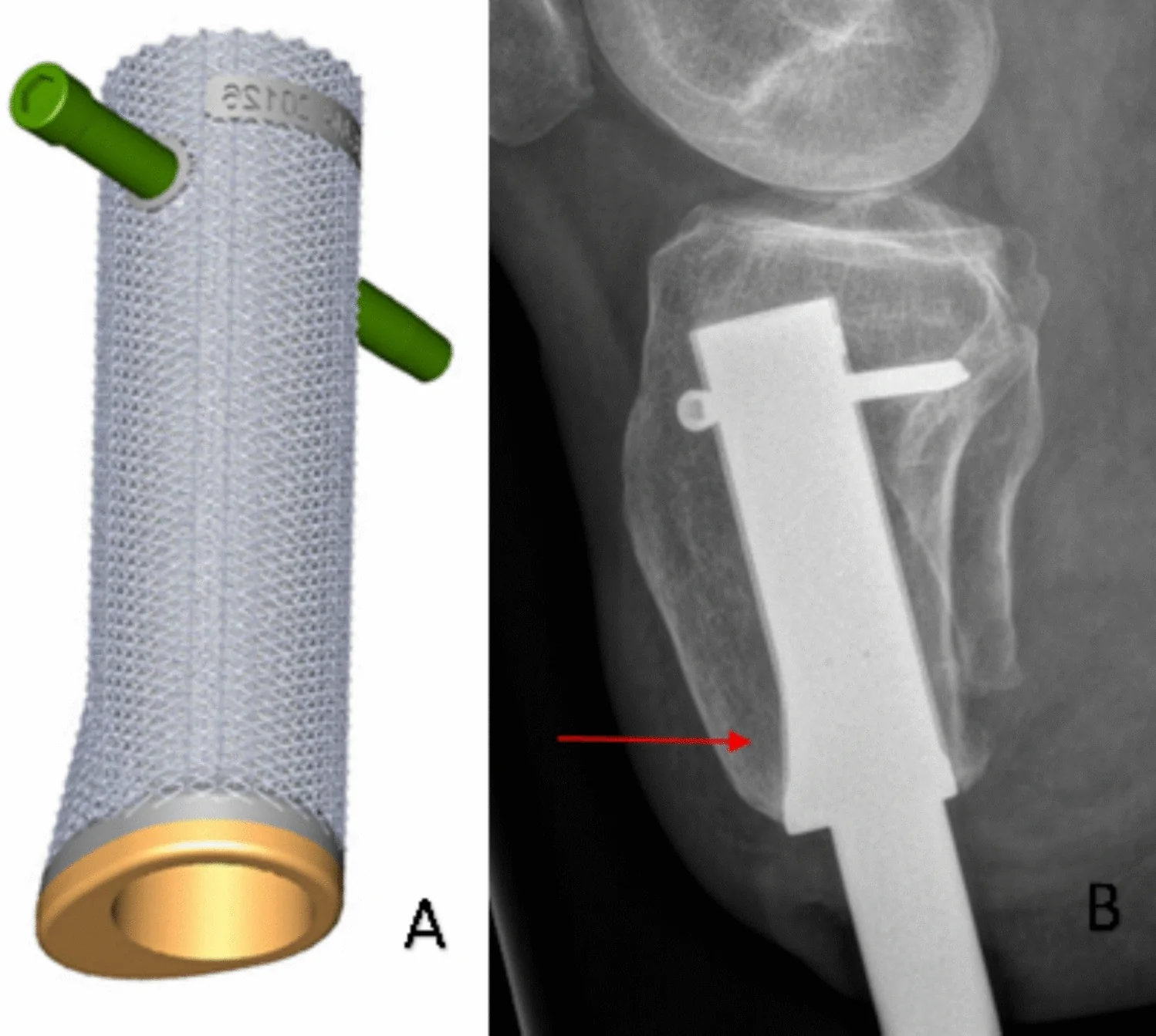
Implant system 4. **A** 3D rendering of implant design with 3D lattice structure completely covering implant. **B** Lateral radiograph showing implant position with arrow pointing to distal 3D lattice structure covering part of the drop

Implant system 4 was used for 9 individuals with 9 implants in the period 2020-2021. It was called the osseointegrated tibial implant (OTI) from the implant system “Bone Anchoring Device for Artificial Limbs” (BADAL X, OTN Implants, Nijmegen, The Netherlands). The implant was manufactured using the same principles as for implant system 3, but with a different manufacturing process. The main difference with implant system 3 was the fact that the 3D lattice structure continued over the drop-like extension covering most of the intramedullary part of the implant (Fig. 4A). The previous manufacturing process also made it impossible to consistently cover the full extent of the implant with the lattice structure. The aim of implant system 4 was to increase the area available for osseointegration and also to stimulate osseointegration at the most distal portion of the implant, thus to adequately seal off the intramedullary canal.

#### Implant System 5

**Fig. 5.**
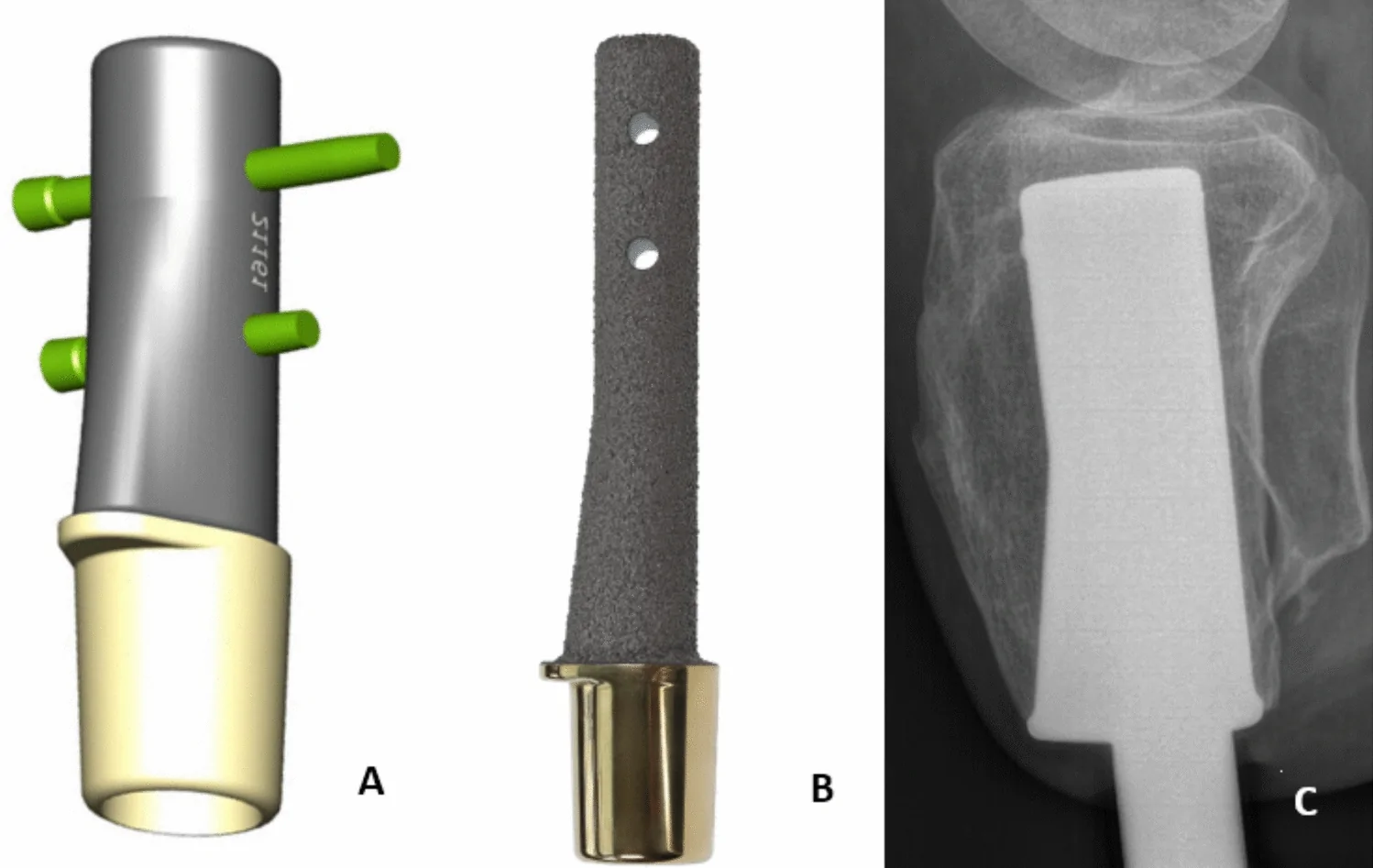
Implant system 5. **A** 3D rendering of implant design with surface coating. **B** Photograph of implant with titanium plasma spray surface coating. **C** Lateral radiograph of implant at 2 years follow-up showing optimal osseointegration

Implant system 5 was used in 51 individuals with 56 implants from 2021, and is currently still in use. Individuals treated between 2021 and 2024 are included in this review. It is an updated version of the OTI from the BADAL X system which was manufactured by CNC milling, instead of the previously used DMLS technique. The DMLS technique was abandoned because the manufacturing process was difficult to validate with regards to product quality and implant strength. Changing the manufacturing process also made it possible to change the titanium alloy type used going from Ti-6Al-4 V to Ti-6Al-7Nb, which is suggested to improve the biocompatibility profile [40]. Furthermore, the 1 mm 3D lattice surface structure was replaced by a 0.35 mm titanium plasma spray (TPS) layer with a 0.05 roughness, increasing porosity by 20-40% (Fig. 5A, B). Although the change from a lattice structure to a TPS-coated surface slightly decreased the surface area of osseointegration, it provided an increase in the net implant core diameter of 1.3 mm. In line with this, the CNC milled implants with TPS surface coated proved to be superior compared to DMLS regarding product quality and mechanical strength in dynamic fatigue tests [41].

## OI-Survival Outcomes

**Fig. 6.**
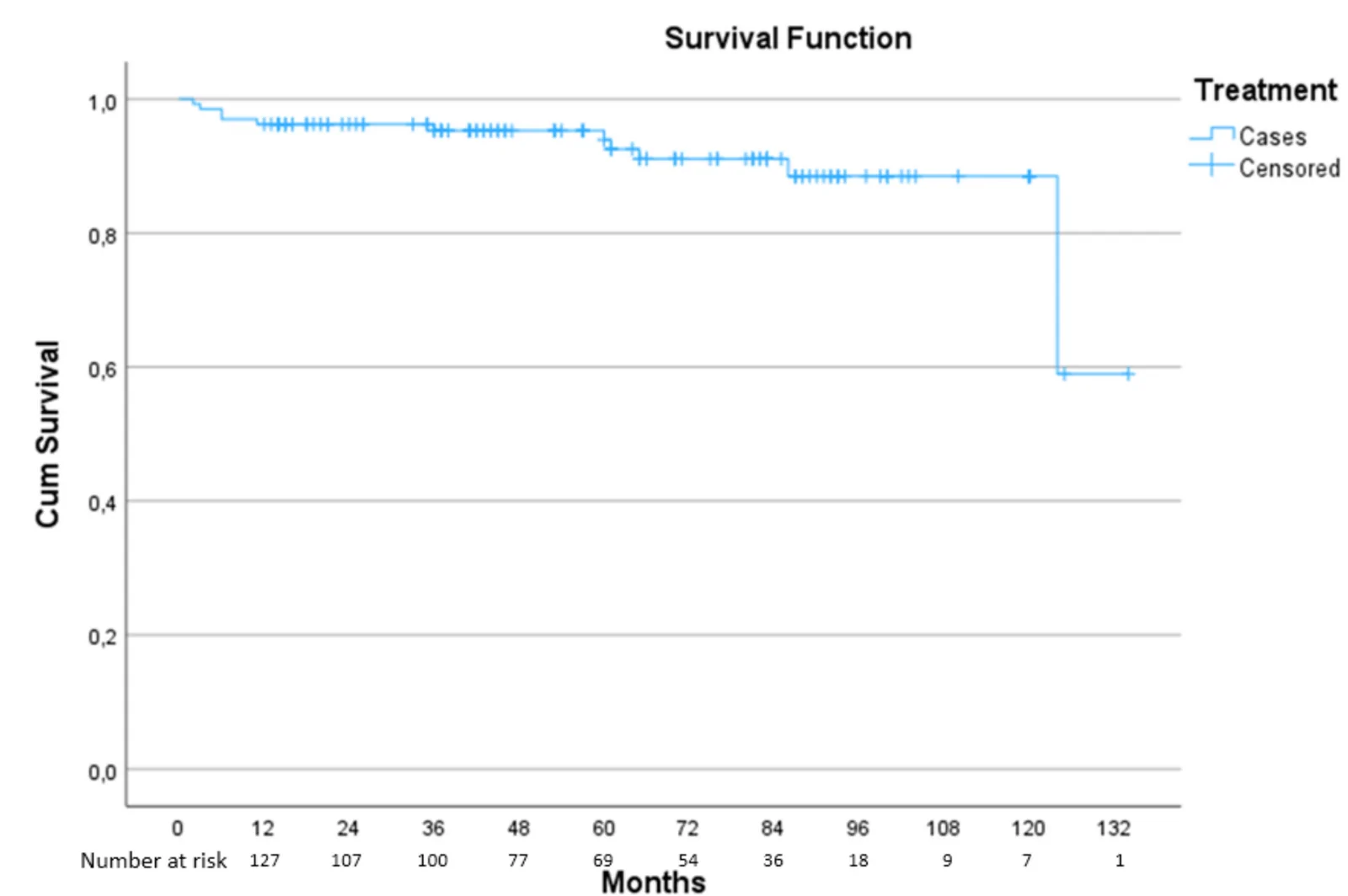
Kaplan–Meier graph of implant survival over time

**Fig. 7.**
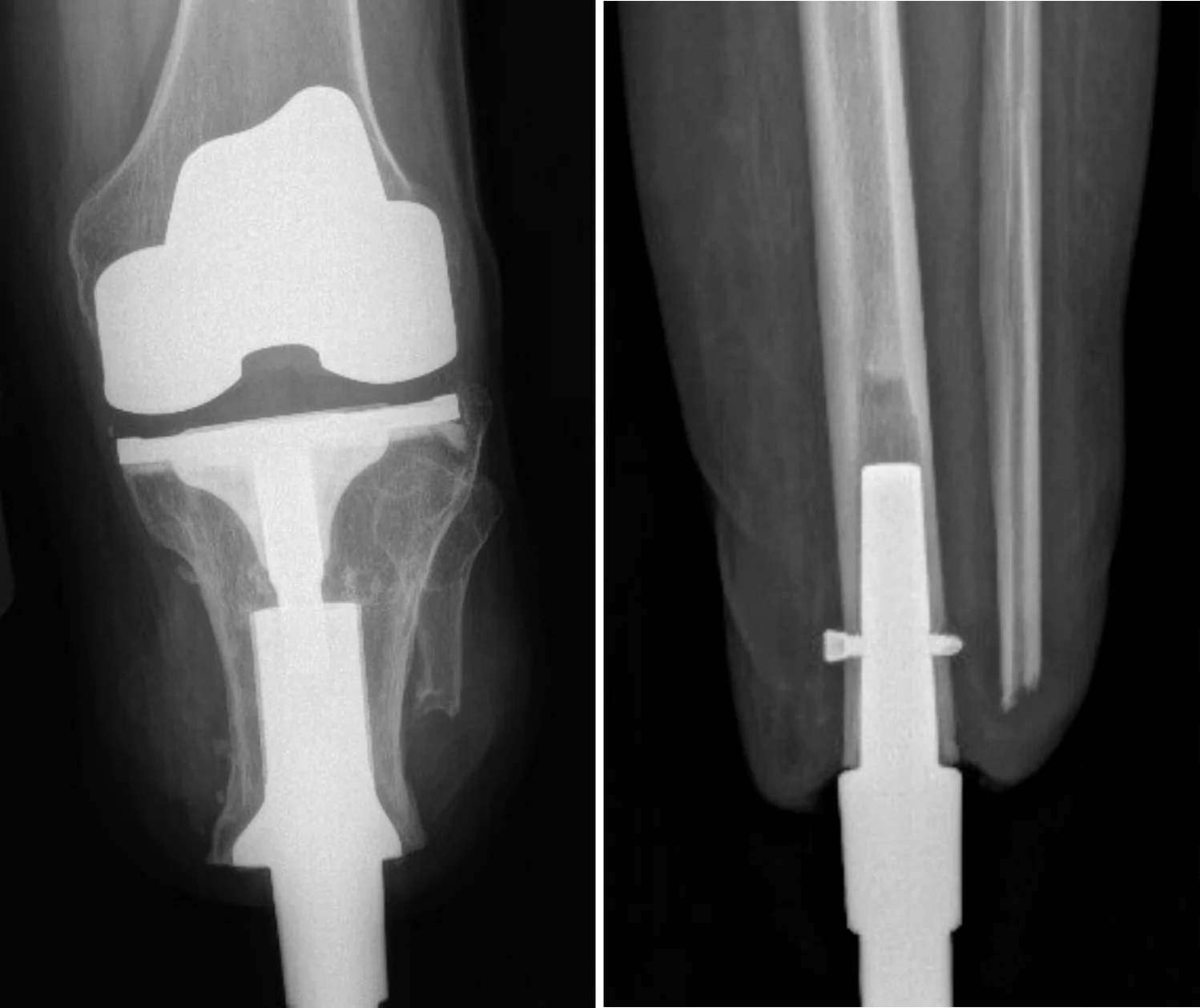
Special implant designs. **A** Radiograph of hollow implant surrounding keel of total knee arthroplasty. **B** Radiograph of implant design for distal tibial amputation with tapered aspect

Implant survival at two, five, and 10 years was 96.2%, 93.9%, and 88.5%, respectively. Implant survival for the total cohort was calculated using the Kaplan–Meier method (Fig. 6). A total of 11 implants failed or were removed out of 133 implants that were followed for a mean of 58 months (Table 3). As mentioned in previous sections, two individuals experienced implant breakages, the sole individual treated with design 1 at 60 months follow-up, and 1 individual treated with a design 3 implant at 65 months follow-up. Two individuals requested a more proximal amputation due to chronic persistent complaints of pain arising from their knee joint. Additional analysis could not provide an implant-related answer or solution for their chronic complaints. One of the individuals had been treated with a design 3 implant which was hollow and customized to permit a total knee arthroplasty just proximal (Fig. 7), and the other individual had undergone traumatic TTA also resulting in ligamentous injury of the knee and was treated with an ultra-short design 4 implant (46 mm length). The latter underwent through-knee amputation and is using a SSP, while the other individual underwent successful TFA combined with a femoral BAP. Five individuals experienced early failure (within 12 months) due to different reasons. Three out of these five individuals experienced early septic loosening of a design 3 implant. Out of these three, one individual was an insulin-dependent diabetic experiencing early surgical site infection with formation of an abscess, eventually undergoing transfemoral amputation after five  months and passing away a couple months later for reasons unrelated to the BAP treatment. The second individual was a vascular patient who refused to stop smoking and required a TFA six  months after BAP treatment due to progression of his vascular disease. The third individual had previously undergone amputation due to complex trauma involving osteomyelitis. It was believed the amputation had taken place proximal to the region of infection and no cultures were taken during primary OI surgery. However, early multi-bacterial septic loosening occurred. The other two individuals that experienced early failure suffered from a lack of osseointegration, both were treated with design 5 implants. The first individual was treated with a special tapered implant suited for a distal tibial amputation. However, the lack of a patient-specific conical reamer led to periprosthetic fracture, and loosening within two  months (Fig. 6B). The second individual underwent BAP treatment in a tibia which had previously undergone radiotherapy, a formal contra-indication for treatment. No implant osseointegration occurred and the implant loosened. The final two implant failures were also septic implant failures. However, these occurred after a longer period of time, in one individual treated with a design 2 implant (failure at 124 months), and one individual treated with a design 5 implant (failure at 61 months). Both individuals experienced distal bone resorption, leading to complaints, for which multiple minor revision surgeries took place, ultimately leading to major septic loosening.

**Table 3.** Implant failure data

Failure	Design	Time of failure	Reason for failure	Dimensions (width x length)^a^	Remark
1	1	60 months	Stem breakage	11 x 105	Thin cobalt–chromium design prone to breakage
2	2	124 months	Septic loosening	21 x 130	Chronic stoma problems due to distal stem coating at stoma level requiring multiple soft tissue revisions
3	3	3 months	Septic loosening	22 x 90	Insulin-dependent diabetes, early post-surgical abscess
4	3	7 months	Septic loosening	21 x 85	Vascular disease, did not refrain from smoking, required transfemoral amputation for disease progression
5	3	86 months	Patient request	24 x 54	Short tibial implant with painful knee arthroplasty
6	3	65 months	Stem breakage	22 x 75	3D printed, no coating on drop showing no distal OI
7	3	11 months	Septic loosening	17 x 80	Post-fracture infection, no cultures taken at implantation. Early infection with candida albicans
8	4	36 months	Patient request	30 x 46	Ultra-short tibial implant, painful knee
9	5	61 months	Septic loosening	16 x 120	Distal bone resorption requiring minor revisions
10	5	2 months	Lack of OI	Tapered 15 > 10 X 60	Distal-tapered implant, early periprosthetic fracture
11	5	6 months	Lack of OI	15 x 100	Previously irradiated bone

*OI* osseointegration

^a^In millimeters

## Recommendations (Expert opinion)

### Strong

- In general, we recommend using patient-specific implant designs for tibial BAP treatment, optimizing implant position, and fixation.
- Consider shortening the residual bone to make room for a larger diameter implant in patients with a narrow intramedullary canal. Where possible, aim for a core diameter of at least 15 millimeters.
- Use implants in which the intramedullary portion is completely covered with an additive surface treatment (i.e., coating), aiming for an increased surface area available for osseointegration, while also stimulating the distal bone-implant interface.

### Moderate

- Consider the use of transverse locking screws for primary stability, especially in (very) short metaphyseal located implants without much cortical contact.
- Aim for a distal seal of the medullary cavity using drop-like implant extensions.
- Consider the use of a relatively thin additive surface treatment maintaining a larger core implant diameter, as aseptic loosening rarely occurs.
- Consider the implant manufacturing process: CNC milling might be preferable to 3D printing with regards to reliability and implant strength properties.
- Metaphyseal located implants might be favorable to diaphyseal or distal implants due to: 1. Possibility of using implants with a larger surface area for osseointegration area, 2. Non-inferiority regarding functionality (i.e., no functional gain from a more distal implant), 3. More experience and standardized surgical instruments for metaphyseal implants.
- Beware of placing the OI to distal since the prosthetist will require a minimum installation height for componentry.

## Data Availability

The authors confirm that the data supporting the findings of this study are available within the article.

## References

[1] Branemark, R., P. I. Branemark, B. Rydevik, and R. R. Myers. Osseointegration in skeletal reconstruction and rehabilitation: a review. *J. Rehabil. Res. Dev.* 38(2):175–181, 2001. 11392650

[2] Hagberg, K., and R. Branemark. One hundred patients treated with osseointegrated transfemoral amputation prostheses–rehabilitation perspective. *J. Rehabil. Res. Dev.* 46(3):331–344, 2009. 19675986

[3] Branemark, P. I., R. Adell, T. Albrektsson, U. Lekholm, S. Lundkvist, and B. Rockler. Osseointegrated titanium fixtures in the treatment of edentulousness. *Biomater.* 4(1):25–28, 1983. 10.1016/0142-9612(83)90065-0 6838955

[4] Hoellwarth, J. S., K. Tetsworth, M. A. Akhtar, and Muderis M. Al. The clinical history and basic science origins of transcutaneous osseointegration for amputees. *Adv. Orthop.* 2022:7960559, 2022. 10.1155/2022/7960559. PMC8956382 35340813

[5] Thesleff, A., R. Branemark, B. Hakansson, and M. Ortiz-Catalan. Biomechanical characterisation of bone-anchored implant systems for amputation limb prostheses: a systematic review. *Ann. Biomed. Eng.* 2018. 10.1007/s10439-017-1976-4. PMC5809556 29327257

[6] Li, Y., and R. Branemark. Osseointegrated prostheses for rehabilitation following amputation : the pioneering Swedish model. *Unfallchirurg.* 120(4):285–292, 2017. 10.1007/s00113-017-0331-4. PMC5371647 28229193

[7] Premarket approval (PMA) OPRA implant system US department of health and human services 2019. https://www.accessdata.fda.gov/scripts/cdrh/cfdocs/cfpma/pma.cfm?id=P190009. Accessed 14 Nov 2025

[8] Aschoff, H. H., A. Clausen, and T. Hoffmeister. The endo-exo femur prosthesis–a new concept of bone-guided, prosthetic rehabilitation following above-knee amputation. *Z. Orthop. Unfall.* 147(5):610–615, 2009. 10.1055/s-0029-1185893. 19938359

[9] Aschoff, H. H., A. Clausen, K. Tsoumpris, and T. Hoffmeister. Implantation of the endo-exo femur prosthesis to improve the mobility of amputees. *Oper. Orthop. Traumatol.* 23(5):462–472, 2011. 10.1007/s00064-011-0054-6. 22083046

[10] Aschoff, H. H., and D. L. Juhnke. Evaluation of 10 years experience with endo-exo femur prostheses–background, data, and results. *Z. Orthop. Unfall.* 150(6):607–614, 2012. 10.1055/s-0032-1327932. 23171987

[11] Juhnke, D. L., J. P. Beck, S. Jeyapalina, and H. H. Aschoff. Fifteen years of experience with Integral-Leg-Prosthesis: cohort study of artificial limb attachment system. *J. Rehabil. Res. Dev.* 52(4):407–420, 2015. 10.1682/JRRD.2014.11.0280. 26348827

[12] Knezevic, A., T. Salamon, M. Milankov, S. Ninkovic, M. Jeremic Knezevic, and Todorovic S. Tomasevic. Assessment of quality of life in patients after lower limb amputation. *Med. Pregl.* 68(3–4):103–108, 2015. 10.2298/mpns1504103k 26214989

[13] Taylor, C. E., H. B. Henninger, and K. N. Bachus. Cortical and medullary morphology of the tibia. *Anat. Rec. (Hoboken).* 304(3):507–517, 2021. 10.1002/ar.24479. PMC8396369 32585072

[14] Atallah, R., R. A. Leijendekkers, T. J. Hoogeboom, and J. P. Frolke. Complications of bone-anchored prostheses for individuals with an extremity amputation: a systematic review. *PLoS ONE.*13(8):e0201821, 2018. 10.1371/journal.pone.0201821. PMC6084937 30092081

[15] Dillingham, T. R., L. E. Pezzin, and E. J. MacKenzie. Limb amputation and limb deficiency: epidemiology and recent trends in the United States. *South Med. J.* 95(8):875–883, 2002. 10.1097/00007611-200208000-00018 12190225

[16] Ziegler-Graham, K., E. J. MacKenzie, P. L. Ephraim, T. G. Travison, and R. Brookmeyer. Estimating the prevalence of limb loss in the United States: 2005 to 2050. *Arch. Phys. Med. Rehabil.* 89(3):422–429, 2008. 10.1016/j.apmr.2007.11.005. 18295618

[17] Frolke, J. P. M., G. M. C. Rommers, A. W. de Boer, T. D. Groenveld, and R. Leijendekkers. Epidemiology of limb amputations and prosthetic use during COVID-19 pandemic in the Netherlands. *Arch. Phys. Med. Rehabil.* 105(2):280–286, 2024. 10.1016/j.apmr.2023.07.012. 37541358

[18] Dudek, N. L., M. B. Marks, S. C. Marshall, and J. P. Chardon. Dermatologic conditions associated with use of a lower-extremity prosthesis. *Arch. Phys. Med. Rehabil.* 86(4):659–663, 2005. 10.1016/j.apmr.2004.09.003. 15827914

[19] Dillingham, T. R., L. E. Pezzin, E. J. MacKenzie, and A. R. Burgess. Use and satisfaction with prosthetic devices among persons with trauma-related amputations: a long-term outcome study. *Am. J. Phys. Med. Rehabil.* 80(8):563–571, 2001. 10.1097/00002060-200108000-00003 11475475

[20] Pezzin, L. E., T. R. Dillingham, E. J. Mackenzie, P. Ephraim, and P. Rossbach. Use and satisfaction with prosthetic limb devices and related services. *Arch. Phys. Med. Rehabil.* 85(5):723–729, 2004. 10.1016/j.apmr.2003.06.002 15129395

[21] Atallah, R., E. Rutjes, J. P. M. Frolke, and R. A. Leijendekkers. Safety and performance of contemporary press-fit titanium osseointegration implants in lower extremity amputation: a five-year follow-up study. *Bone Jt. J.* 107(486):94, 2025. 10.1302/0301-620X.107B4.BJJ-2024-0754.R1. 40164180

[22] Reetz, D., R. Leijendekkers, M. J. Edwards, and J. P. Frolke. Past, present, and future of bone-anchored prosthesis for individuals with lower limb amputation in The Netherlands. *Inj.*56(Suppl 1):112534, 2025. 10.1016/j.injury.2025.112534. 41173552

[23] Gstoettner, C., S. Salminger, A. Sturma, V. Moser, T. Hausner, R. Branemark, et al. Successful salvage via re-osseointegration of a loosened implant in a patient with transtibial amputation. *Prosthet. Orthot. Int.* 45(1):76–80, 2021. 10.1177/0309364620953985. 33834747

[24] Thouvenin, C., M. Bertrand-Marchand, R. Klotz, M. Puges, T. Fabre, and A. Delgove. Bone-anchored prostheses for lower limb amputation in a French cohort with 1-15 years of follow-up: implant survival rates, mechanical complications, and reported outcomes. *Eur. J. Orthop. Surg. Traumatol.* 34(2):885–892, 2024. 10.1007/s00590-023-03738-0. 37750975

[25] Aschoff, H. H., and D. L. Juhnke. Endo-exo prostheses: osseointegrated percutaneously channeled implants for rehabilitation after limb amputation. *Unfallchirurg.* 119(5):421–427, 2016. 10.1007/s00113-016-0175-3. 27142510

[26] Hoffmeister, T., F. Schwarze, and H. H. Aschoff. The endo-exo prosthesis treatment concept: improvement in quality of life after limb amputation. *Unfallchirurg.* 120(5):371–377, 2017. 10.1007/s00113-017-0350-1. 28470463

[27] Akhtar, M. A., J. S. Hoellwarth, S. Al-Jawazneh, W. Lu, C. Roberts, and Muderis M. Al. Transtibial osseointegration for patients with peripheral vascular disease: a case series of 6 patients with minimum 3-year follow-up. *JB JS Open Access.* 6:2, 2021. 10.2106/JBJS.OA.20.00113. PMC8238302 34235362

[28] Atallah, R., J. J. Li, W. Lu, R. Leijendekkers, J. P. Frolke, and Muderis M. Al. Osseointegrated transtibial implants in patients with peripheral vascular disease: a multicenter case series of 5 patients with 1-year follow-up. *J. Bone Jt. Surg. Am.* 99(18):1516–1523, 2017. 10.2106/JBJS.16.01295. 28926380

[29] Atallah, R., H. van de Meent, L. Verhamme, J. P. Frolke, and R. A. Leijendekkers. Safety, prosthesis wearing time and health-related quality of life of lower extremity bone-anchored prostheses using a press-fit titanium osseointegration implant: a prospective one-year follow-up cohort study. *PLoS ONE.*15(3):e0230027, 2020. 10.1371/journal.pone.0230027. PMC7062258 32150598

[30] Haidary, A., J. S. Hoellwarth, K. Tetsworth, A. Oomatia, and Muderis M. Al. Transcutaneous osseointegration for amputees with burn trauma. *Burns.* 49(5):1052–1061, 2023. 10.1016/j.burns.2023.02.006. 36907716

[31] Leijendekkers, R. A., G. van Hinte, J. P. Frolke, H. van de Meent, F. Atsma, M. W. Nijhuis-van der Sanden, et al. Functional performance and safety of bone-anchored prostheses in persons with a transfemoral or transtibial amputation: a prospective one-year follow-up cohort study. *Clin. Rehabil.* 33(450):64, 2019. 10.1177/0269215518815215. PMC6416705 30537856

[32] Muderis, M. A., Y. C. Tan, W. Lu, K. Tetsworth, D. Axelrod, R. Haque, et al. Transtibial osseointegration following unilateral traumatic amputation: an observational study of patients with at least two years follow-up. *Inj.*55(6):111568, 2024. 10.1016/j.injury.2024.111568. 38669890

[33] Panzures, A. C., M. A. Akhtar, J. S. Hoellwarth, K. Tetsworth, and Muderis M. Al. Transcutaneous osseointegration for amputees as a result of sepsis management: a case series of nine patients with a mean follow-up of 4 years. *Strategies Trauma Limb Reconstr.* 20(1):17–24, 2025. 10.5005/jp-journals-10080-1639. PMC12445137 40979932

[34] Reif, T. J., N. Khabyeh-Hasbani, K. M. Jaime, G. A. Sheridan, D. M. Otterburn, and S. R. Rozbruch. Early experience with femoral and tibial bone-anchored osseointegration prostheses. *JB JS Open Access.* 6:3, 2021. 10.2106/JBJS.OA.21.00072. PMC8416017 34497971

[35] Saleib, M. M., E. M. M. Van Lieshout, D. Verduin, H. P. De Graaff, M. A. Paping, M. H. J. Verhofstad, et al. Activities of daily living in lower limb amputees with a bone-anchored prosthesis: a retrospective case series with 24 months’ follow-up. *Acta. Orthop.* 94:499–504, 2023. 10.2340/17453674.2023.19670. PMC10573365 37830879

[36] van Vliet-Bockting, C., R. Atallah, J. P. M. Frolke, and R. A. Leijendekkers. How to improve patient selection in individuals with lower extremity amputation using a bone-anchored prosthesis. *Clin. Orthop. Relat. Res.* 483(6):1075–1092, 2025. 10.1097/CORR.0000000000003369. PMC12106247 39853312

[37] Vinson, A. L., N. W. Vandenberg, M. E. Awad, C. L. Christiansen, J. W. Stoneback, and B MMG. The biomechanical influence of transtibial bone-anchored limbs during walking. *J. Biomech.*168:112098, 2024. 10.1016/j.jbiomech.2024.112098. PMC11151175 38636112

[38] Hoellwarth, J. S., K. Tetsworth, A. Oomatia, M. A. Akhtar, H. Xu, and Muderis M. Al. Association between osseointegration of lower extremity amputation and mortality among adults. *JAMA Netw. Open.*5(10):e2235074, 2022. 10.1001/jamanetworkopen.2022.35074. PMC9561949 36227599

[39] Mohamed, J., D. Reetz, H. van de Meent, H. Schreuder, J. P. Frolke, and R. Leijendekkers. What are the risk factors for mechanical failure and loosening of a transfemoral osseointegrated implant system in patients with a lower-limb amputation? *Clin. Orthop. Relat. Res.* 2021. 10.1097/CORR.0000000000002074. PMC8923606 34889879

[40] Mitra, K., A. K. Movva, M. O. Sohn, J. M. Tennyson, G. M. Talaski, S. B. Adams, et al. Alloy selection and manufacturing technologies for total ankle arthroplasty: a narrative review. *Mater. (Basel).* 18:16, 2025. 10.3390/ma18163770. PMC12387489 40870087

[41] Baleani, M., L. Cristofolini, and M. Viceconti. Endurance testing of hip prostheses: a comparison between the load fixed in ISO 7206 standard and the physiological loads. *Clin. Biomech. (Bristol).* 14(5):339–345, 1999. 10.1016/s0268-0033(98)00085-0. 10521611

